# Intravital imaging reveals new ancillary mechanisms co-opted by cancer cells to drive tumor progression

**DOI:** 10.12688/f1000research.8090.1

**Published:** 2016-05-16

**Authors:** Claire Vennin, David Herrmann, Morghan C. Lucas, Paul Timpson

**Affiliations:** 1 The Kinghorn Cancer Centre, Cancer Division, The Garvan Institute of Medical Research, Sydney, NSW, Australia; 2 St Vincent’s Clinical School, Faculty of Medicine, University of New South Wales, Sydney, NSW, Australia

**Keywords:** Intravital imaging, tumor progression

## Abstract

Intravital imaging is providing new insights into the dynamics of tumor progression in native tissues and has started to reveal the layers of complexity found in cancer. Recent advances in intravital imaging have allowed us to look deeper into cancer behavior and to dissect the interactions between tumor cells and the ancillary host niche that promote cancer development. In this review, we provide an insight into the latest advances in cancer biology achieved by intravital imaging, focusing on recently discovered mechanisms by which tumor cells manipulate normal tissue to facilitate disease progression.

## Introduction

Within tumors, intricate crosstalk between cancer cells and the surrounding microenvironment supports cancer initiation and progression. Importantly, these interactions not only shape the development of the primary tumor but also are required at secondary sites to develop a microenvironment permissive to metastatic growth. This appreciation of the dynamic cancer-stroma interactions in tumor progression has led to a transition from traditional *in vitro* assays to more complex *in vivo* models to faithfully embrace the intricacy of cancer. In these models, intravital imaging has been used to dissect the molecular events governing cancer progression and revealed unprecedented information on the behavior of cells in their native environment, both within primary tumors and at distant metastatic sites. In combination with traditional and static approaches used in cancer research, intravital imaging has significantly expanded our understanding of the complexity of cancer biology, as it allows us to directly image the spatiotemporal dynamics of cancer progression in live settings and at the whole-organ, cellular, subcellular, and molecular levels. Here, we outline the latest discoveries facilitated by intravital imaging of tumors, which could not otherwise be achieved *in vitro,* and discuss how intravital imaging techniques used in other disease contexts could be repurposed for cancer research.

## Imaging the tumor vasculature to study cancer growth and dissemination

Cancer cells use and co-opt the surrounding stroma to promote their expansion and dissemination. Tumor-associated blood vessels serve a dual function during cancer development: they provide tumor tissue with essential oxygen and nutrients but can also act as carriers for circulating cancer cells. While various models have been developed *in vitro* for studying microvasculature systems ^1, 2^, several intravital imaging tools, such as fluorescent proteins or nanoparticles, have been employed to understand the interactions between cancer cells and blood vessels in live settings, an aspect that cannot be recapitulated *in vitro* ^3– 6^. For example, fluorescently labeled lectins, such as *Lens culinaris* agglutinin, have been developed to selectively bind to endothelial cells ^3, 4^. Intravenous co-injection of agglutinin and fluorescently labeled cancer cells was recently used with intravital imaging of the embryonic chicken chorioallantoic membrane (CAM). This is a surrogate assay for the study of cancer cell extravasation and metastatic colonization and has recently provided insight into the time course and molecular mechanisms involved in cancer cell extravasation ^3, 4^. Here, the authors demonstrated that intravascular cancer cells initially move along the luminal endothelium in an amoeboid manner and subsequently start to extend protrusions in-between endothelial cells into the extravascular stroma. Cancer cells then gradually push through endothelial cell-cell junctions, in some cases even displacing endothelial cells ^4^. Interestingly, invadopodia marker proteins such as cortactin or MT1-MMP were shown to be localized at the protrusions of extravasating cancer cells, and knockdown of these markers impaired cell extravasation and the formation of metastatic colonies within the CAM ^4^. In line with this, tail-vein injection of invadopodia-deficient cells into mice resulted in a decreased number of lung metastases, showing that invadopodia are a key feature for transendothelial cancer cell migration ^4^. Similarly, providing intravascular cancer cells in the CAM assay with hyaluronic acid, which is a component of the extracellular matrix (ECM) overexpressed in many tumor types ^7– 9^, led to increased cell extravasation ^3^. Using this probe therefore demonstrated that the ECM not only represents a physical barrier against chemotherapy delivery ^10^ but can also be co-opted by cancer cells for secondary site colonization.

**Figure 1.**
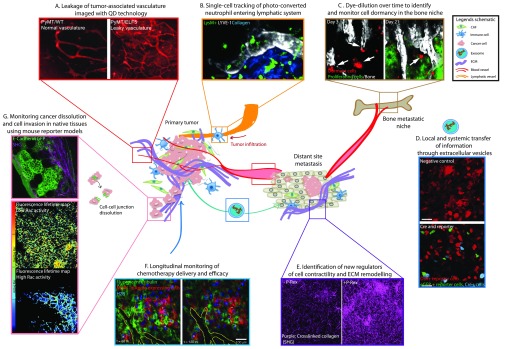
Intravital imaging of the ancillary mechanisms promoting cancer progression. **A**. Quantum dot (QD) imaging of angiogenesis and leaky tumor vasculature. Adapted from 13. **B**. Tracking of photo-converted neutrophils migrating through lymphatic networks. Adapted from 38. **C**. Identification of dormant myeloma cells homed into the bone niche. Adapted from 48. **D**. Direct visualization of local and systemic transfer of extracellular vesicles and exosomes between different cellular compartments. Adapted from 60. **E**. Second harmonic generation (SHG) analysis of collagen crosslinking to identify new regulators of extracellular matrix (ECM)-driven aggressiveness. Adapted from 74. **F**. Longitudinal monitoring of chemotherapy pharmacokinetics and targeting through an optical window. Adapted from 93. **G**. Dissection of spatiotemporal dynamics of molecular events driving cancer invasion and dissolution in native tissues using reporter mice. Adapted from 96 and 103. Abbreviations: CAF, cancer-associated fibroblast; GFP, green fluorescent protein.

Fluorescent dextrans and quantum dot (QD) nanoparticles are also commonly used to visualize blood vessels. More recently, they have been employed during *in vivo* cancer research to investigate vascular integrity in live tumor tissues ^11, 12^. For example, intravital time-course imaging of QDs in the MMTV-PyMT mouse model of breast cancer allowed Ormandy and colleagues to demonstrate that cancer cells at the primary site can use the surrounding blood vasculature during the progression of invasive and metastatic cancer ^13^. Here, ELF5 induction resulted in QD leakage and accumulation within the interstitial space, in line with the formation of hemorrhagic tumors, and increased the number of lung metastases ( Figure 1A, blood vessels in red, note the signal in areas around leaky vessels in PyMT/ELF5 mice ^13^). Furthermore, we previously used QDs to map the vascularization of pancreatic subcutaneous xenograft tumors and showed *in vivo* that Src activity and drug-mediated Src inactivation in cancer cells correlate with their position relative to intratumoral blood vessels ^11^. New organic QDs with improved biocompatibility, stability, and two-photon absorption cross-section and fluorescently tagged viral nanoparticles have recently been developed for real-time intravital mapping of the blood vasculature *in vivo* ^14, 15^. Fluorescently labeled red blood cells and mice with fluorescently labeled endothelial cells were also engineered to mark the blood vasculature *in vivo* ^16– 18^, and together with QDs and dextran, these approaches are likely to improve our understanding of how the tumor-associated vasculature supports cancer progression.

The lymphatic system presents another route of cancer cell dissemination. For example, Das *et al.* recently used intravital imaging of melanoma cells injected into the mammary fat pad with a lymphatic tracer dye to visualize cancer cell migration towards tumor-draining lymph nodes ^19^. The authors demonstrated that lymphatic sinuses of the lymph nodes, but not peripheral lymphatics, secrete CCL8, which activates CCR1 on the cancer cell compartment to permit entry into the lymph node ^19^. Inhibition of CCR1 did not impair primary tumor cells from entering the lymphatic system but blocked their exit into the lymph node and arrested the circulating cells within nearby lymphatic vessels. This indicates that lymphatic cancer cell dissemination not only occurs through passive transport but also is supported by paracrine chemotactic signaling ^19^. Recently, a transgenic reporter of lymphatic vessels was generated by crossing LSL-tdTomato mice with Prox1-Cre-ERT2 mice to induce fluorescent reporter expression in lymphatic vessels and map cell trafficking within lymphatic networks ^20^. While the authors used this model to track dendritic cells entering tdTomato-expressing lymphatic vessels upon acute inflammation *in vivo*, this technology could be repurposed for future use in cancer research to watch circulating tumor cells disseminating through the lymphatic system.

## Imaging the tumor-associated immune system

During cancer progression, tumor cells interact with and manipulate the immune system to facilitate their growth and escape from cell death ^21– 24^. Here, dual intravital imaging of leaky blood vessels and immune cells has been used to identify novel mechanisms of drug action. In particular, the antitumor activity of bisphosphonates, which are commonly used in skeletal diseases, such as osteoporosis ^25^, has been explored in a 4T1 mammary tumor mouse model ^26^. Here, it was demonstrated that fluorescently labeled bisphosphonates enter the primary tumor site via the leaky vasculature and bind to microcalcifications within the tissue. Bisphosphonates were then incorporated by tumor-associated macrophages (TAMs), and the authors suggest that this might impair the function of TAMs ^26^, which have been shown to potentiate tumor progression ^27^. Thus, intravital imaging allowed the authors to delineate an unknown mechanism of action underlying the partial benefits of bisphosphonate treatment in breast cancer patients. In line with this, TAMs have also been identified in the tumor microenvironment of metastasis (TMEM), a hotspot of vascular permeability characterized by the physical interaction among tumor cells, TAMs, and endothelial cells ^28^. Intravital imaging of blood vasculature using QDs and fluorescent dextran demonstrated that Tie2 ^hi^-expressing TAMs induce transient vascular leakage via vascular endothelial growth factor A (VEGFA) signaling, thereby facilitating tumor cell intravasation. Interestingly, intravital imaging also showed that transendothelial migration preferentially occurred in regions containing a TMEM, and specifically targeting this site could slow down the spread of cancer cells ^28^. Intravital imaging has also been used recently to compare the motility of two immune effector cell types, natural killer (NK) cells and cytotoxic T-lymphocytes (CTLs) ^29^. Here, the authors showed that while NK cells form short and fast contacts with tumor cells independent of calcium signaling, CTLs form long-lasting contacts with tumor cells that require calcium influx into CTLs. Interestingly, both cell types were shown to require calcium influx for efficient killing ^29^, thus intravital imaging has provided new insights into the cell-type-specific interactions between tumor cells and the immune system which are needed for specific and efficient killing of tumor cells to occur and which cannot be mimicked faithfully *in vitro*.

On a similar note, using intravital imaging, Moalli *et al.* characterized a potential pathway through which tumor-derived antigen is locally processed and presented to generate a systemic immune response ^30^. Injection of B16.F10 melanoma cells, transfected with tdTomato as a surrogate tumor-derived antigen, into the mouse footpad led to a robust immune response after 30 days measured by high titers of anti-B16.F10-tdTomato IgG ^30^. Imaging of tumor-draining lymph nodes showed that tdTomato was localized in macrophages in the lymphatics and also in follicular dendritic cells (FDCs). Using mouse models of macrophage, FDC, or B-cell deficiency, the authors suggest that tdTomato is taken up by macrophages and subsequently localizes with FDCs, which are then scanned by B-cells, generating a systemic immune response ^30^. Similarly, intravital imaging of mCherry-expressing mammary tumors was used to characterize antigen presentation at the tumor site ^31^. Here, the authors showed that a subset of myeloid cells function as tumor dendritic cells by taking up and presenting tumor-derived antigen to CTLs. Interestingly, it was shown that CTLs engage with the antigen-presenting cells and subsequently become arrested in this engagement, potentially inhibiting cytolytic effects and tumor rejection. The authors suggest that tumor immunotherapy may help release the CTL blockade following their initial attraction and clustering with antigen-presenting cells at the tumor site ^31^. Similarly, Boissonas *et al.* showed by intravital imaging that infiltrating T-lymphocytes can become trapped with tumor dendritic cells following chemotherapy, suggesting that tumor dendritic cells can function as a sink that retains the T-cell-driven anti-tumor immune response ^32^. These findings enhance our understanding of the immune response to cancer development both at primary and metastatic sites, thus intravital imaging may help pave the way to the development of new presentation-derived therapeutics. For example, intravital imaging in the MMTV-PyMT mouse model of breast cancer enabled the identification of a type of macrophage-dendritic cell (M-DC) ^33^. Here, the authors used fluorescent dextran to label M-DCs and revealed that the depletion of M-DCs decreases tumor growth and metastasis and could therefore present a promising future target ^33^. Similarly, recent intravital imaging of CTL intratumoral migration *in vivo* revealed that treatment with anti-CD137 mAb prolongs the interaction between CTLs and cancer cells, thereby improving the antitumor CTL activity ^34^. This is in parallel with recent improvements in anti-PD1 immunotherapy to educate the immune system to recognize and kill melanoma cells ^35^ and may represent a promising approach for the improvement of immune-based therapeutics in multiple cancer types.

Several fluorescent reporter systems to specifically label and track immune cells within native tissues have been developed recently. One such example is the Catchup mouse model ^36^, wherein a bicistronic cassette of Cre and tdTomato was engineered to be expressed under the neutrophil-specific locus Ly6G. Using this tool, the authors tracked neutrophil migration and studied neutrophil-specific gene functions *in vivo*. Similarly, a transgenic mouse with ubiquitous expression of Kikume, a green fluorescent protein (GFP) that can be irreversibly photo-converted to red via violet light excitation, was used to characterize dendritic cell ^37^ and neutrophil migration ^38^ *in vivo* ( Figure 1B, single-cell tracking of neutrophils ^38^). For example, upon bacterial infection, neutrophils were recruited to the mouse ear and were photo-converted. Further intravital imaging allowed the authors to track the photo-converted cells and to identify and map new patterns of neutrophil migration and homing into the lymph nodes ^38^. Another example is the MacGreen transgenic reporter mouse in which c-fms-driven GFP expression is targeted to macrophages, trophoblasts, and granulocytes ^39^. Using this reporter, Pai *et al.* could track circulating leukocytes during cerebral malaria pathogenesis and identified plasmodium-specific CD8+ T lymphocytes as important regulators of this disease ^40^. Crossing these reporter animal models with mouse models of cancer may provide much-needed insights into how cancer cells manipulate and interact with the host immune system to promote tumor progression. For example, intravital imaging may allow us to identify the migration routes of immune cells during cancer progression and treatment or to characterize tumor immune subtypes ^41, 42^ in a dynamic manner to better inform on immunotherapies in cancer, a feature of cancer progression that cannot be modeled or assessed accurately *in vitro*.

## Cancer-fibroblast interactions facilitate tumor progression

Fibroblasts are also frequently recruited to tumor sites, and intravital imaging has recently been used to show that stromal fibroblasts can regulate overall tumor response to chemotherapy. For example, intravital imaging of a xenograft model of melanoma through an optical window demonstrated paradoxical activation of cancer-associated fibroblasts (CAFs) at the primary site upon treatment with Braf inhibitors, thereby leading to increased stiffening of the ECM compartment ^43^. Here, the authors show that the stiffened ECM in turn activated adhesion-mediated signaling pathways, such as focal adhesion kinase (FAK), to promote cancer cell survival and so decrease anti-Braf treatment efficacy ^43^. Interestingly, impairing cellular adhesion tension through FAK inhibition reduced the safe haven provided by the ECM and resulted in a significantly enhanced response to chemotherapy ^43^.

Similarly, Lee *et al.* used *in vivo* whole-body bioluminescence imaging to investigate the mechanisms of resistance to anti-insulin-like growth factor (anti-IGF) treatment with cixutumumab ^44^, which recently showed limited efficacy in clinical trials ^45, 46^. Here, the authors demonstrated that upon treatment with cixutumumab, cancer cells underwent a protective reprogramming characterized by increased production of IGF, which in turn led to the recruitment and activation of host fibroblasts to the primary site through IGF-2R-dependent paracrine signaling. Activated fibroblasts were then shown to secrete pro-angiogenic factors and to potentiate metastasis and disease relapse ^44^. Their findings also correlated with changes in the tumor microenvironment of patients undergoing anti-IGF-based therapies ^44^, suggesting that preventing fibroblast activation can impair extrinsic mechanisms of chemoresistance and in turn improve cixutumumab efficacy.

Intravital imaging has also been used to implicate stromal fibroblasts in cancer cell dormancy, an important cause of disease relapse, which remains poorly understood ^47^. In a recent study, Lawson *et al.* developed an approach for longitudinal intravital imaging of myeloma cells that had lodged within dense bone tissue ^48^. Using a dye-dilution system, the authors were able to label and track slowly proliferating, dormant cells over time and showed that these cells preferentially lodge in direct contact with the endosteal bone surface and interact with host bone cells to establish a protective niche for persistence of metastasis (see Figure 1C showing dormant cells [red] lodged at the surface of the bone [white] and proliferating cells [green] ^48^). Importantly, this study demonstrated that cell dormancy is a reversible state, which can be turned “off” and “on” by signals emanating from host osteoblasts and osteoclasts. The authors suggest that using bone-active drugs to either prevent the reactivation of dormant cells or take cells out from dormancy before treating them with chemotherapy could improve outcome and reduce disease relapse. Their findings present new opportunities for the treatment of metastatic cancers with a bone tropism such as breast, prostate, or kidney cancer ^48^.

Tumor cells have also been shown to co-opt fibroblasts to promote cancer spread, and recent *in situ* imaging of secondary organ colonization has been used to reveal how host fibroblasts are implicated in the preparation of the metastatic niche ^49^. In an orthotopic model of breast cancer, dual monitoring of metastatic initiating breast cancer cells and lung fibroblasts recently revealed that reciprocal interactions between the two cell types are required for successful establishment of lung metastases. Here, the authors demonstrate that a biphasic intercellular crosstalk progressively modifies both compartments. Briefly, activation of a fibrotic program in the fibroblast population led to the initiation of a mesenchymal-to-epithelial transition within the cancer cell compartment, promoting the onset of metastasis ^49^. Taken together, these studies suggest that preventing fibroblast activation and reprogramming driven by tumor cells during cancer progression may help improve sensitivity to chemotherapy and impair cancer dissemination. Intravital imaging of secondary sites prone to metastasis is required to directly visualize the stages of metastatic colonization; however, long-term and deep imaging of metastatic organs remains technically challenging. Optical imaging windows now enable longitudinal and deep imaging of events occurring at both primary sites and secondary organs such as the liver, spleen, kidney, and pancreas ^50, 51^. For instance, monitoring the early events promoting the formation of the liver metastatic niche was achieved using optical imaging windows ^52^. Interestingly, this study demonstrated that single extravasated cells are initially highly motile and proliferative and form pre-micrometastases within the liver before merging into micrometastases with reduced migration. Importantly, impairing early tumor cell migration significantly reduced the metastatic burden ^52^. Similarly, the formation of the liver metastatic microenvironment and response to chemotherapy has been studied using longitudinal scanning multiphoton microscopy in an intrasplenic model of colorectal cancer ^50^ and informed on the response of metastatic cells to chemotherapy.

One limitation to longitudinal intravital imaging is the movement of live tissue, such as heart or lung, which can perturb high-resolution intravital imaging, and current research focuses on motion compensation systems to correct for tissue movement ^53– 55^. Additionally, the probability of imaging an invasive or metastatic event can be considered very low; however, optogenetic intravital imaging can be used to specifically activate or inhibit signaling pathways and subsequently trigger such an event.

## Intercellular crosstalk is supported by extracellular vesicles

Reciprocal interactions between cancer and stromal cells require an exchange of information, for example, in the form of physical cell-cell interaction or secretion of protein ligands or extracellular vesicles (EVs). Direct visualization of signal transmission between cancer cells and the host environment has long been technically challenging; however, the recent development of new intravital imaging tools has helped elucidate the mechanisms which enable intercellular exchange of information within both primary and metastatic sites. Recent studies showed that cancer cells can manipulate host stromal cells through the local and systemic transfer of exosomes and other EVs ^56– 58^. For instance, live imaging of a chicken embryo model of fibrosarcoma indicated that exosomes promote cancer cell-ECM adhesion assembly and are required for persistent cell movement ^59^. In addition, elegant *in situ* studies demonstrated that exosomes secreted from primary cancer cells localize at secondary metastatic sites, fuse with host cells, and can “educate” secondary organs through the activation of fibrotic and inflammatory programs ^56, 57^. In line with this study, direct intravital imaging of exosome secretion and uptake by different cell populations ^60, 61^ has recently been achieved. Here, Zomer *et al.* developed a Cre-loxP-based system whereby cells that take up Cre(+) EVs exhibit a change in color (see Figure 1D showing Cre[+] cells [blue], reporter cells that incorporated Cre[+] EVs [green], and reporter cells that did not ^60^). With this approach, the authors demonstrated that EVs are locally and systemically transferred from aggressive cancer cells to less malignant cancer cells within the same mouse to coordinate a whole-body systemic metastatic program *in vivo* and to enhance the overall metastatic ability of the tumor ^60, 61^. Recent findings also demonstrate that mitochondrial DNA (mtDNA) can be transferred from host stromal cells to cancer cells. Here, the authors genetically manipulated melanoma and breast cancer cells to lack mtDNA and showed *in situ* that they can acquire host mtDNA to restore mitochondrial functions such as bioenergetic dynamics and respiration. In addition, incorporation of host mtDNA by cancer cells led to increased tumor growth and metastatic spread, demonstrating that tumor cells can take up material from the host to drive cancer aggressiveness ^62, 63^. Interestingly, Osswald *et al.* used *in vivo* multiphoton scanning of astrocytomas over a year to study a novel mechanism of intercellular connection ^64^, wherein tumor cells in astrocytomas use ultra-long protrusions (>500 μm in length) to form a network of multi-cellular communication. The authors show that this network supports tumor cell invasion and proliferation and protects cancer cells against radiotherapies ^64^. Future developments in intravital imaging to directly visualize mechanisms of cell-cell interaction may help us understand how cancer cells and host tissues interact and together drive cancer aggressiveness, and in the future may help us to uncouple this support mechanism.

## Imaging biomechanical properties of the extracellular matrix

Over the last decade, intensive work has shown that ECM abundance and features, such as stiffness, topography, and porosity, contribute to tumor cell proliferation, invasion, and chemoresistance ^65– 72^. Multiphoton imaging technologies, such as second harmonic generation (SHG) imaging ^73^, have been used to study the modulation of ECM characteristics during cancer progression and have recently identified new regulators of biomechanical tissue properties (see Figure 1E showing collagen deposition in *in vitro* 3D organotypic fibroblast-collagen matrices upon induction of P-Rex, a guanine exchange factor for Rac1 ^74^). For instance, the actin cytoskeleton and a number of its regulatory proteins, such as the family of Rho GTPases, are known to govern the remodeling of the ECM ^69, 75– 77^, and live SHG imaging has provided new insights into the tight regulation of Rho GTPases *in vivo*. In collaboration with Samuel and colleagues, we helped identify the intracellular signaling protein 14-3-3 ζ as a novel negative regulator of Rho-kinase-driven ECM stiffening in wound healing ^78^. Here, live monitoring of stromal fibroblasts coupled with SHG imaging of collagen fibers in a 3D *in vitro* environment was used to show that loss of 14-3-3 ζ in dermal fibroblasts impairs their ability to remodel collagen and to stiffen the ECM, and such effects were also seen in *ex vivo* animal samples ^78^. Interestingly, this study also showed in squamous cell carcinoma that 14-3-3 ζ is downregulated while collagen deposition is increased, suggesting that ROCK signaling is no longer restrained and controlled *in vivo* in this disease ^69, 78, 79^. These findings suggest new therapeutic options for re-purposing 14-3-3 ζ-based treatments to facilitate uniformed and controlled wound healing ^78^, which in future can also be used in the context of cancer to re-establish normal tissue homeostasis of solid tumors ^69, 80, 81^.

The hypoxic tumor environment was also recently identified as a novel regulator of the ECM ultrastructure and stiffness at both primary and metastatic sites ^82, 83^. For example, hypoxia was shown to inhibit prolyl hydroxylase domain protein 2 (PHD2) in CAFs *in vitro* and *in vivo,* leading to a reversion of CAF activation and a decrease in their ability to remodel and stiffen the ECM ^82^. In addition, proteins secreted from hypoxic tumors were demonstrated to prepare and activate the ECM of future metastatic sites. One such example is the enzyme lysyl oxidase (LOX), which is upregulated in bone tropic breast cancer cells ^83^. *In vivo,* injection of LOX resulted in the formation of osteolytic lesions and in turn provided circulating breast cancer cells with a focal pre-metastatic niche for bone colonization, whereas LOX targeting with a blocking antibody partially reverted this phenotype and decreased tumor burden, as visualized by whole-body *in vivo* imaging ^83^. Intravital SHG imaging has also been used to directly monitor changes of the ECM structure and content during cancer progression in live settings. For example, Walsh *et al.* performed intravital SHG imaging of breast cancer xenografts to study the effects of trastuzumab treatment on the ECM ^84^. Interestingly, their study demonstrated that trastuzumab induces changes in collagen density and alignment and thereby identified a non-cellular response to drug treatment ^84^. In addition, longitudinal SHG imaging of orthotopic breast xenografts was performed at the tumor margin to identify the key components of the tumor microenvironment that modulates cell invasion ^85^. Here, the authors used dual imaging of single cells and collagen fibers and showed that slow-locomotion breast cancer cells form invadopodia to remodel and stiffen the collagen matrix while they move through the surrounding tissue. Further use of intravital SHG imaging will provide important insights into the role of the ECM and its manipulation during tumor progression.

Rapidly developing intravital technologies and tools have provided unprecedented insights into cancer complexity and have also opened up promising avenues for pre-clinical cancer research. For example, SHG imaging has recently been translated to study the properties of the ECM in biopsy samples of cancer patients. For instance, in the context of pancreatic cancer, we recently used SHG imaging to analyze the properties of the ECM in a human pancreatic tissue microarray (>80 samples) and detected a positive correlation between fibrillar collagen abundance, tumor stage, lymph node spread, and vascular invasion, suggesting that fine-tuned targeting of collagen crosslinking may be a valid approach in this disease ^86^. In line with this study, biophysical analysis of collagen remodeling was conducted in human breast cancer biopsies (>20 samples) and revealed a progressive increase of collagen reorganization and orientation as invasive cancer lesions develop ^87^. Similarly, in a cohort of matched human breast tissues with areas of high and low mammographic density from the same patient (>15 samples), we analyzed ECM organization by gray-level co-occurrence matrix (GLCM), an image texture-based approach used to quantify the organization of collagen fiber networks ^88^. In this study, Britt and colleagues demonstrated that increased deposition and crosslinking of collagen fibers correlates with a high risk of developing breast cancer ^88^. Taken together, these studies along with our knowledge from intravital animal models indicate that upon acquisition of human biopsies, characterization of ECM abundance and crosslinking using medium-throughput SHG imaging can potentially be used as a biomarker to predict patient prognosis and tumor stage. Moreover, the development of new probes to directly image distinct components of the ECM such as hyaluronan ^3, 89^, elastin ^90^, or fibronectin ^91^ is likely to facilitate our understanding of the role of the ECM in cancer development and may help us improve therapies targeting specific components of the ECM.

## New tools for live, intravital, and *in situ* imaging in translational cancer research

Fluorescence-based biosensors are emerging as a reliable tool to dissect mechanisms of drug target activity and chemoresistance. In a large panel of MEK inhibitor (MEKi)-resistant cell lines, live imaging of extracellular signal-regulated kinase (Erk) and S6K fluorescent biosensors was used to interrogate the molecular bases of resistance to MEKi in Kras-mutant and Braf-mutant cancer cell lines *in vitro*. Here, the authors showed that upon treatment with MEKi, Erk and PI3K pathways can maintain mTORC1 activity and in turn promote cell growth, thereby leading to resistance to treatment ^92^. Furthermore, fluorescently tagged drugs were used to decipher mechanisms of drug resistance. For instance, a fluorescent analog of eribulin was designed to monitor *in vivo* drug pharmacokinetics. Here, the authors engineered a portion of tumor cells to express multidrug-resistance 1 (MDR1)-mApple fusion protein, and dual imaging of eribulin and MDR1-mApple demonstrated that resistance to eribulin was directly correlated with vascular architecture and MDR1-mediated drug efflux. This study also demonstrated that inhibition of MDR1 reversed the multidrug-resistant phenotype and improved eribulin efficacy ^93^ ( Figure 1F showing fluorescent eribulin [green] diffusing into tumor tissue and being incorporated only by cancer cells not expressing MDR1-mApple fusion protein at 1 and 2 hours following treatment with eribulin ^93^).

Taking these approaches further, for the assessment of whole-tissue or whole-body protein activity, transgenic mice have been generated with ubiquitous or Cre-inducible expression of reporters of the activity of Erk, PKA, Rac1, cAMP, or cell cycle progression ^94– 99^ ( Figure 1G, map of Rac activity where red and yellow mark low Rac activity and blue and green highlight high Rac activity ^96^). Crossing these biosensor mice with animal models of cancer allows us to dissect the spatiotemporal mechanisms of the molecular events driving cancer in native tissues. For example, Kumagai *et al.* recently crossed their Erk biosensor mouse with a mouse model of mammary tumor formation and described a stable heterogeneous pattern of Erk activity ^100^. Interestingly, the authors showed that cells with low Erk activity were more successful in forming tumor spheres *in vitro* and expressed high markers of mammary cancer stem cells compared to cells with high Erk activity ^100^. These findings indicate that tumor heterogeneity in Erk activity may be beneficial to establish cancer stem cells with low Erk activity for self-renewal, whereas high Erk activity supports rapid growth and expansion ^100^. Similarly, Erk pulsing and propagation dynamics emanating from the skin and hair follicle have also recently been demonstrated *in vivo* ^101^ and have significant implications for future research in melanoma ^102^.

Another recent example of biosensor mouse imaging in cancer is our E-cadherin-GFP mouse, which enabled us to perform photobleaching experiments *in vivo* to monitor cell-cell junction dynamics ( Figure 1G, E-cadherin-GFP expression in cell-cell junctions of the lactating mammary gland ^103^). Here, we used fluorescence recovery after photobleach (FRAP) and fluorescence loss in photobleach (FLIP) imaging along with kymograph analysis to quantify E-cadherin mobility of healthy and diseased tissue. Using this approach, we could correlate high E-cadherin mobility in the cell membrane with a decrease in junction strength and integrity and an increase in cell invasiveness ^103^. Crossing this mouse with a genetically engineered model of pancreatic cancer allowed us to recapitulate the full spectrum of pancreatic cancer progression and to dissect the genetic stages of early cancer dissolution. This mouse was also used as a tool for drug discovery by testing new anti-invasive agents, such as dasatinib, in pancreatic cancer, where we demonstrated that dasatinib is able to strengthen cell-cell junction integrity in line with decreased invasiveness in this setting ^103^. This is in line with current clinical assessment in patients and its known effectiveness in the KPC pancreatic cancer mouse model ^104^.

Mouse models with ubiquitous expression of molecular reporters can now be used to monitor events in a large range of organs and diseases; however, due to light scattering within tissues, the imaging depth with current two-photon lasers is limited to several-hundred μm, limiting intravital imaging of large organs and tumors. Current multiphoton setups include three-photon lasers in combination with optical parametric oscillators (OPOs) that increase the excitation wavelength, as red and near-infrared light can penetrate deeper into tissue and reduces tissue scattering ^105, 106^. This requires the use of fluorescent proteins that can be excited with near-infrared wavelengths, such as iRFP or iRFP variants, which have been recently described ^107, 108^. Furthermore, computational models, such as adaptive optical correction, have been used to overcome light scattering and to increase imaging depth ^109^. Another imaging technique, called photoacoustic tomography (PAT), makes use of the phenomenon that photon absorption forms pressure waves that can be detected. Although PAT increases imaging depth, it strongly reduces image resolution, which can be partially overcome by the use of near-infrared fluorescent proteins ^110, 111^. Furthermore, current proceedings to reduce the distal optics and mechanical components of intravital microscopes ^112– 114^ should move the field towards intravital endoscopy of cancer biology ^115^.

Lastly, optogenetics, which rely on the inherent properties of light to observe and accurately control complex biochemical and signaling events ^116^, is emerging as another promising tool to interrogate cancer dynamics. Optogenetics allows researchers to manipulate gene expression or the activity of cellular signaling pathways and has recently enabled photocontrol of proteins with subcellular resolution. These approaches have been used to modify protein conformation ^117^, stimulate DNA binding ^118^, control enzymatic ^119^ or receptor tyrosine kinase activity ^120^, induce protein localization ^121^, manipulate protein-protein interactions ^122^, and study signaling pathway events ^123, 124^. For instance, optogenetics was used to demonstrate that localized Rac1 activity is sufficient to produce precisely regulated cell protrusion events to control directed cell motility *in vivo* ^125– 128^. Most importantly, optogenetics may enable us to modulate cancer-related signaling pathways in a defined spatiotemporal manner and allow us to follow the fate of de-regulated cells in their *in situ* environment. In summary, intravital imaging has demonstrated its capacity to allow us to explore the ancillary mechanisms supporting cancer progression and provide novel routes for the development of future therapeutics to target and impair these support mechanisms.

## Abbreviations

CAF, cancer-associated fibroblast; CAM, chorioallantoic membrane; CTL, cytotoxic T-lymphocyte; ECM, extracellular matrix; ERK, extracellular signal-regulated kinase; EV, extracellular vesicle; FAK, focal adhesion kinase; FDC, follicular dendritic cell; FLIP, fluorescence loss in photobleaching; FRAP, fluorescence recovery after photobleaching; GLCM: gray-level co-occurrence matrix; IGF, insulin-like growth factor; LOX, lysyl oxidase; M-DC, macrophage-dendritic cell; MDR1, multi-drug resistance 1; MEKi, MEK inhibitor; mtDNA, mitochondrial DNA; NK cell, natural killer cell; OPO, optical parametric oscillator; PAT, photoacoustic tomography; PHD2, prolyl hydroxylase domain protein 2; QD, quantum dot; SHG, second harmonic generation; TAM, tumor-associated macrophage; TMEM, tumor microenvironment of metastasis; VEGFA, vascular endothelial growth factor A.
